# Horizontal Spatial Metaphors for Morality: A Cross-Cultural Study of Han Chinese Students and Ethnic Minority Hui Students in China

**DOI:** 10.3389/fpsyg.2018.01145

**Published:** 2018-07-05

**Authors:** Rui Chen, Jiao Sai, Qi Zhu, Renlai Zhou, Peng Li, Shunchao He

**Affiliations:** ^1^School of Educational Science and Management, Yunnan Normal University, Kunming, China; ^2^Dehong Nationality No.1 Middle School, Dehong, China; ^3^Behavioural Science Institute, Radboud University, Nijmegen, Netherlands; ^4^School of Social and Behavior Sciences, Nanjing University, Nanjing, China

**Keywords:** spatial metaphor, moral, culture, Han Chinese, Hui Chinese, cross-culture

## Abstract

Metaphor is a bridge for understanding abstract concepts (the target domain) from concrete concepts (the source domain). This study, with two experiments, aimed to investigate the cultural differences of the horizontal spatial metaphors for morality between two groups of students: Han Chinese, the ethnic majority, and Hui Chinese, an ethnic minority in China. Experiment 1 adopted a spatial Stroop task. It showed that neither Hui nor Han students exhibited horizontal spatial metaphors for morality. Experiment 2 adopted a modified implicit association test paradigm to enhance the association between the moral concepts and the horizontal spatial positions. In Experiment 2, we found horizontal spatial metaphors for morality in Hui students, but not in Han students. These results indicated that the differences of horizontal spatial metaphors between Hui and Han participants were influenced by the different cultures they live in. Moreover, this study also found that the association between the source domain and the target domain was an important factor for metaphor formations.

## Introduction

Metaphor is a kind of rhetoric in linguistics. Its interpretation reflects as much on the interpreter as on the originator (Davidson, 1978). Metaphor allows people to think abstractly. For example, “TIME IS MONEY.” Time is an abstract concept, which could be difficult to understand without a reference to a physical object, like “money,” which can be understood clearly and directly. In this sense, metaphor is a hidden link between abstract concepts and concrete concepts. This implies that understanding of abstract concepts could be enhanced by utilizing concrete concepts as support. Lakoff and Johnson (1999) indicated that physical metaphors are useful to represent abstract concepts in most psycholinguistic research.

Metaphors commonly exist in our conceptual systems, which influences our thoughts and behavior, and is reflected in our languages (Lakoff and Johnson, 1980). Moral cognition, as a part of the complex system of conceptual metaphors, is also largely metaphorical. This enhances the understanding of moral principles and values (Lakoff, 2002; Johnson, 2014). The acquisition of moral cognition depends on individual experiences and the cultural environment that they live in Gibbs (1999, 2006, 2014) and Kovecses (2015). Further research suggested that we develop our moral metaphors based on our subjective experiences in certain social-cultural environments, and we distinguish these metaphors into moral and immoral concepts (Yu, 2016). In most existing research, the subjective experiences mainly included light and dark (Meier and Clore, 2004), clean and dirty (Rozin et al., 1986; Haidt and Joseph, 2006; Schnall and Harvey, 2008), and up and down (Lakoff and Johnson, 1999; Lan, 1999). A recent study, moreover, indicated that even in thinking about other people, spatial metaphor also plays a key role (Gozli et al., 2018). In particular, light, clean, up and superior states were associated with morality; whereas dark, dirty, down, and inferior states were associated with immorality.

Among these subjective experiences, research on spatial metaphors mainly investigates vertical spatial metaphors for morality, compared to other spatial metaphors, since we have direct and profound understanding for vertical spatial concepts with our daily experience of gravity (Gibbs, 2006; Jia, 2014, unpublished). Popular paradigms on this topic include the spatial Stroop task (Meier et al., 2007) and the implicit association test (IAT; Meier and Robinson, 2004; Meier et al., 2007). The spatial Stroop task, also called the categorization task, required participants to categorize words as moral or immoral when each word appeared in the upper half or lower half of the screen (Meier and Robinson, 2004; Li and Xu, 2012). In the IAT, participants first determined whether a prime stimulus was at the top or bottom of a computer screen, and then whether the word appeared on the center of the screen had a moral or immoral meaning (Greenwald et al., 1998; Meier and Robinson, 2004; Jia, 2014, unpublished). Most research using these paradigms found that the moral words (e.g., upright) were categorized faster when the prime stimuli were presented toward the top of the screen, whereas the negative words (e.g., low-minded) were categorized faster when the prime stimuli presented toward the bottom of the screen (Lakoff and Johnson, 1999; Meier et al., 2007; Hill and Lapsley, 2009; Wang and Lu, 2013; Jia, 2014, unpublished). This revealed that the vertical spatial metaphors for morality were stable across different cultures.

There were a small amount of studies on the horizontal spatial metaphors. Casasanto (2009) conducted both horizontal and vertical tasks with 28 left-handers and 191 right-handers to verify a body-specificity hypothesis (BSH): spatial metaphors for morality were influenced by the dominant hand. The results in the horizontal task showed that right-handers tended to associate rightward space with positive stimuli and leftward space with negative stimuli, whereas left-handers showed the opposite pattern, associating rightward space with negative stimuli and leftward with positive stimuli. Nevertheless, the results in the vertical task showed that both right-handers and left-handers applied the mental metaphors “GOOD IS UP” and “BAD IS DOWN” (Casasanto, 2009). These results revealed that handedness affected the horizontal spatial metaphors for morality, but did not affect the vertical spatial metaphors. Lakoff and Johnson (1980) emphasized the influence of culture on the metaphors. They demonstrated that the metaphor formation is closely related to the cultural environment people live in. Kita and Essegbey (2001) found that the use of the left hand is a taboo in Ghana. Specifically, giving, receiving, eating, and drinking with the left hand are considered rude by Ghanaian people. Similarly, to Hui people, who believe in Islam, the right side is superior, whereas the left side is inferior. Chapter 69, Verse 19 of the Holy Quran describes, “As for him who is given his record in his right hand, he will say: take, read my book! Surely I knew that I should have to meet my reckoning. Then he will be in blissful state... But as for him who is given his record in his left hand, he will say: Oh, would that I had not been given my book. And knew not what my reckoning! Oh, would that it had been death! My wealth hath not availed me. My power hath gone from me” (Ma, 2013). An additional investigation before experiment showed that Muslims have to use their right hands while gifting to or accepting from others, even step in with their right foot first while going home or visiting others. Therefore, to Muslims, the right hand is blessed, but the left hand is not. In traditional Han culture, the superiority or inferiority in interpersonal relationships is reflected by horizontal spatial positions as well. However, different from Hui culture, the traditions in Han culture were often changed in different dynasties throughout history, and varied in diverse situations. For example, from the perspective of the official titles, during the Spring and Autumn Period, and from the Wei and Jin to the Qing Dynasty, those with “left” in the name were superior to those with “right” in the name (e.g., the left grand councilor was superior to the right grand councilor.). On the contrary, during the Pre-Qin Period, the Western Han Dynasty, the kingdom of Shu Han, and the Yuan Dynasty, titles containing the word “right” were superior to those containing the word “left” (Zhang, 1987; Sun, 2010). In terms of seat positions, however, the tradition that superiors always sit on the left has been observed since ancient times (Zhang, 1987). Overall, in contrast to the unchanging rules of “right-superior and left-inferior” in Hui culture, rules in Han culture have been changed a number of times.

In summary, the influence of the culture on metaphors is unavoidable (Lakoff and Johnson, 1980). Large volumes of research from different cultures corroborate the consistency of vertical spatial metaphors for morality (Lakoff and Johnson, 1999; Hill and Lapsley, 2009; Wang and Lu, 2013; Jia, 2014, unpublished). However, there were few empirical studies to verify the horizontal spatial metaphors for morality across different cultures. Previous research was mostly focused on descriptive phenomenon, and empirical evidence would provide us with scientific explanations of the cultural influences on the spatial metaphors for morality, thus promote our understanding for cultures of different ethnic groups. In addition, empirical research on this topic would support to advance the theories in spatial metaphors of morality, and thus enrich the theories of metaphor formations.

This study was to investigate whether there are cross-cultural differences on the horizontal spatial metaphors for morality in Hui and Han middle school students. Specifically, we hypothesized that (1) Hui students would show horizontal spatial metaphors for morality, valuing right as superior and left as inferior; (2) Han students would not show horizontal spatial metaphors. These hypotheses reflected the moral influence of different cultures from Hui and Han ethnics on the horizontal spatial metaphors for morality.

## Experiment 1 Spatial Stroop Task

We adopted the spatial Stroop task to test whether the participants respond in different speeds to moral or immoral words when the words were presented on the left or right parts of the screen. If the hypotheses were verified, Hui students would respond faster to moral words when they were presented on the right compared on the left, and faster to immoral words when they were presented on the left compared on the right. However, Han students would not show any differences of respond time when the words were presented either left or right.

### Methods

#### Participants

Hui people in Yunnan Province postured as “big mixed and small settlement” for generations. According to records, Qutong Village is a typical Islamic Hui village with an intact traditional Hui culture. In order to exclude the influence of extraneous variables, such as climate, terrain and genetics, we chose a group of 60 consisted of 30 Han students and 30 Hui students in the same area. They are from Qutong Middle School at Qutong Village, in Yongping County, Dali Bai Autonomous prefecture, Yunnan Province. The Each ethnic group was made up of 15 males and 15 females. The mean age of Hui students was 16.70 ± 0.54. The mean age of Han students was 15.83 ± 0.59. All participants were physically healthy, with normal or corrected-to-normal vision, and right-handed. They participated in the study voluntarily, and received gifts as a reward.

This study was carried out in accordance with the recommendations of Ethical Principles of Psychologists and Code of Conduct from the American Psychological Association with written informed consent for all participants’ guardians. An Ethics Commission at Yunnan Normal University approved the study.

#### Material

We chose 120 Chinese words related to morality based on middle school textbooks. Among them, half of words had a moral meaning and the other half had an immoral meaning. We also included another 40 words with no moral alignment. All the 160 words were two-character Chinese words.

10 middle school students (five Hui and five Han), who did not participate in the following experiments, evaluated the moral valence of these words on a nine-point scale, from 1 (most immoral) to 9 (most moral). Neutral words, or words irrelevant to morality, were scored as approximately 5. After evaluation, from the 120 words related to morality, we selected the 30 words with the highest score as the moral group of words, and the 30 words with the lowest score as the immoral group of words. Also, from the 40 words without moral alignment, we chose 30 words with scores between 4 and 6 as neutral words. The evaluation of the chosen words’ moral valence is shown in **Table 1**.

**Table 1:** The evaluation of the chosen words’ moral valence (M ± SD).

Word type	Number of words	Moral valence
Moral	30	7.61 ± 0.55
Neutral	30	4.96 ± 0.29
Immoral	30	2.57 ± 0.51

One-way ANOVA suggested that the main effect of word type was significantly different, *F*(2,89) = 869.38, *p* < 0.001. A significant homogeneity of variance test was found before paired comparison of these three types of words, *F*(2,87) = 8.76, *p* < 0.001. Thus, we used Dunnet T3 correction in *post hoc* multiple comparisons, and showed that the valence of words with moral meaning was significantly higher than the valence of neutral words (*p* < 0.001) and immoral words (*p* < 0.001); in addition, the valence of words with immoral meaning was significantly lower than the valence of neutral words, *p* < 0.001. The results showed that the selection of moral words and immoral words was effective.

Thirty selected words in both the moral and the immoral types were applied to the following experiment. The words were presented in black, size 40, Song font, and with a gray background.

#### Device

The words appeared on a 19-inch LCD screen at 1600 × 900 resolution, with a refresh rate of 60 Hz. The computer used a dual core Intel i3 CPU, with a core speed of 3.7 GHz. E-prime 1.0 was used to run the task.

#### Design

The experiment adopted 2 (word valence: moral vs. immoral) × 2 (word position: left vs. right) within-subject design. The dependent variable was reaction time.

#### Procedure

The experiment was carried out individually. Before the experiment was started, the experimenter explained the instructions. After fully understanding the instructions, the participants began a practice block. The practice block used 10 words which were not selected for the experiment. The participants sat at a distance of 60 cm from their computer screen. In each trial, the participants first saw a fixation cue ‘+’ at the center of the screen for 1000 ms, followed by a word either 5 cm to the left or to the right of the fixation cue for 3000 ms. The participants were required to evaluate the word as having a moral or immoral meaning as quickly and accurately as possible. If the word had a moral meaning, the participants were supposed to press ‘A’; or if immoral, ‘L.’ If the participants did not respond within 3,000 ms, a blank gray screen would appear for 1,000 ms, followed by the next word in the experiment. After 10 practice trials, the experimental block started. The procedure of the experimental block was the same as the practice blocks.

The experimental block included 120 trials. Words from the moral group and the immoral group were presented randomly. The frequency and the chance of a word appearing in either position were the same for both word types. The correct response keys of ‘A’ and ‘L’ were reversed for half of the participants, in order to rule out the influence of handedness.

### Results

The correct response accounted for 98.17% of all the trials. We excluded the response times which exceeded M ± 2.431SD. This criterion for excluding an observation as outlier was proposed by Van Selst and Jolicoeur to adjust the number of SD according to sample size (Van Selst and Jolicoeur, 1994; Cousineau and Chartier, 2010; for a review). Total exclusion accounted for 1.64% of all the trials. See the raw data of this experiment in the **Supplementary Material**.

For Hui students (see **Table 2**), a 2 (valence: moral vs. immoral) × 2 (word position: left vs. right) repeated measures analysis of variance (ANOVA) shows that there was a significant main effect of the moral valence, *F*(1,29) = 56.60, *p* < 0.001, $\eta_{p}^{2}$ = 0.64. Hui students responded faster to the words with moral meaning than the words with immoral meaning. However, the main effect of horizontal position was not significant, *F*(1,29) = 0.25, *p* = 0.62, $\eta_{p}^{2}$ = 0.01. The interaction effect between the words’ moral valence and the horizontal positions was not significant, *F*(1,29) = 1.77, *p* = 0.19, $\eta_{p}^{2}$ = 0.06.

**Table 2:** The response time of the spatial Stroop task in Hui students (ms) (M ± SD).

Horizontal positions	Words’ moral valence	
	Moral	Immoral
Left	806 ± 98	854 ± 100
Right	804 ± 92	862 ± 99

For Han students (see **Table 3**), a 2 (valence: moral vs. immoral) × 2 (word position: left vs. right) repeated ANOVA shows that there was a significant main effect of the moral valence, *F*(1,29) = 10.64, *p* < 0.001, $\eta_{p}^{2}$ = 0.27. Han students also responded faster to the words with moral meaning than the words with immoral meaning. The main effect of horizontal position was not significant, *F*(1,29) = 1.45, *p* = 0.24, $\eta_{p}^{2}$ = 0.05. The interaction effect between the words’ moral valence and the horizontal positions was not significant, *F*(1,29) = 0.63, *p* = 0.43, $\eta_{p}^{2}$ = 0.02.

**Table 3:** The response time of the spatial Stroop task in Han students (ms) (M ± SD).

Horizontal positions	Words’ moral valence	
	Moral	Immoral
Left	809 ± 139	824 ± 135
Right	809 ± 138	817 ± 137

### Discussion

Experiment 1 showed that the Han students did not respond at different speeds on horizontal positions of moral or immoral words; that is, they did not have horizontal spatial metaphors for morality. This was in accordance with our second hypothesis. The Han culture does not have unchanged left or right positions for superiors and inferiors. Therefore, the Han students do not have bias toward horizontal positions of moral or immortal words. However, although Hui culture constantly regards the right superior to the left, Hui students also did not show horizontal spatial metaphors for morality, which went against our first hypothesis.

Reviewing previous research, we found that most researches on vertical spatial metaphors for morality were conducted with adults. Yin (2014, unpublished) conducted research with primary school students, middle school students, and adults. He found that the vertical spatial metaphors were stronger in adults than in primary or middle school students. This showed that the spatial metaphor for morality becomes stronger as people grow up. Marmolejo-Ramos et al. (2013), furthermore, suggested that a higher saliency of the vertical plane was found than the horizontal plane in the allocation of valenced words from different languages even at the conceptual level. Thus, we deduced that in our research, the associations between morality and horizontal spatial positions in middle school students were relatively weak. When the spatial Stroop task required the participants to determine the moral valence of the words and to ignore the words’ positions, the students were not fully aware of the horizontal positions. Therefore, Hui students did not exhibit the spatial metaphors for morality. This result indicated that for the participants who had weak associations between moral valence and the horizontal positions, the activation of words’ spatial positions was insufficient.

In order to verify our reasoning, we adopted a modified IAT (Meier and Robinson, 2004; Jia, 2014, unpublished) in Experiment 2. This task strengthened spatial position awareness through the judgment task of dot position, therefore we could better investigate whether there were horizontal spatial metaphors for morality in Hui students, and whether there were cross-cultural differences of horizontal spatial metaphors for morality in Hui and Han students.

## Experiment 2 Modified IAT Task

This experiment adopted a modified IAT task to investigate whether there were cross-cultural differences of horizontal spatial metaphors for morality in Hui and Han middle school students. We used the classic IAT paradigm in a novel way. We utilized a spatial prime to enhance the spatial awareness in participants, thus to strengthen the association between positions and morality, in order to elicit existing spatial metaphors more evidently.

### Method

#### Participants

We chose 60 middle school students from Qutong Middle School at Qutong Village, in Yongping County, Dali Bai Autonomous prefecture, Yunnan Province. The group of 60 consisted of 30 Han students and 30 Hui students. Each ethnic group was made up of 15 males and 15 females. The mean age of Hui students was 16.47 ± 0.57. The mean age of Han students was 15.47 ± 0.59. All participants were physically healthy, with normal or corrected-to-normal vision, and right-handed. They did not participate in the word evaluation task or Experiment 1. They participated in the study voluntarily, and received gifts as a reward.

This study was carried out in accordance with the recommendations of Ethical Principles of Psychologists and Code of Conduct from the American Psychological Association with written informed consent for all participants’ guardians. An Ethics Commission at Yunnan Normal University approved the study.

#### Material and Device

The same as Experiment 1.

#### Design

We adopted 2 (word valence: moral vs. immoral) × 2 (prime position: left vs. right) within-subject design to conducted the study. The dependent variable was reaction time.

#### Procedure

The experiment was carried out individually. Before the experiment started, the experimenter explained the instructions. After fully understanding the instructions, the participants began a practice block. The practice block used 10 words which were not selected for the experiment. The participants sat at a distance of 60 cm from their computer screen. In each trial, the participants first saw a fixation cue ‘+’ at the center of the screen for 1,000 ms, then saw this cue together with a spatial prime in the form of a red circle either 5 cm to the left or to the right of the ‘+’ for 3,000 ms. The participants had as much time as necessary to judge the position of this spatial prime on the screen. If the circle appeared on the left of the ‘+,’ the participants pressed ‘A’; or if on the right, ‘L.’ After that, the fixation and the spatial prime disappeared, and a word appeared at the center of the screen for 3,000 ms. The participants evaluated the word as having a moral or immoral meaning as quickly and accurately as possible. If the word had a moral meaning, the participants were supposed to press ‘A’; or if immoral, ‘L.’ If the participants did not respond within 3,000 ms, a blank gray screen would appear for 1,000 ms, followed by the next word in the experiment.

After 10 trials of practice, the experimental block started. The procedure of the experimental block was the same as the practice blocks. The experimental block included 60 trials. The position of the spatial prime was randomized, and so the chance of it appearing on either side of the fixation ‘+’ was equal. The frequency of the words with a moral meaning or immoral meaning was also the same. The correct response keys of ‘A’ and ‘L’ were reversed for half of the participants, in order to rule out the influence of handedness.

### Results

The accuracy of judging the position of the spatial prime was not counted. The correct response of word evaluation accounted for 98.17% of all the trials. We excluded the response times which exceed M ± 2.431SD. Total exclusion accounted for 0.95% of all the trials. All the data were analyzed by SPSS. See the raw data of this experiment in the **Supplementary Material**.

For Hui students (see **Table 4**), a 2 (valence: moral vs. immoral) × 2 (prime position: left vs. right) repeated measures analysis of variance (ANOVA) showed that the main effect of the words’ valence was significant, *F*(1,29) = 31.43, *p* < 0.001, $\eta_{p}^{2}$ = 0.52, indicating that Hui students evaluated the words with moral meaning faster than those with immoral meaning. The main effect of the prime position was significant, *F*(1,29) = 4.32, *p* = 0.047, $\eta_{p}^{2}$ = 0.13. The valence × position interaction was significant, *F*(1,29) = 716.50, *p* < 0.001, $\eta_{p}^{2}$ = 0.96. Hui students evaluated words with moral meaning faster when the spatial prime appeared on the right, compared to if the prime was on the left; whereas they evaluated the words with immoral meaning faster when the prime appeared on the left, compared to if the prime was on the right.

**Table 4:** The response time of the adjusted IAT task in Hui students (ms) (M ± SD).

Prime positions	Words’ moral valence	
	Moral	Immoral
Left	821 ± 58	585 ± 57
Right	543 ± 51	843 ± 62

For Han students (see **Table 5**), a 2 (valence: moral vs. immoral) × 2 (prime position: left vs. right) repeated ANOVA showed that the main effect of the words’ valence was significant, *F* (1,29) = 6.02, *p* = 0.02, $\eta_{p}^{2}$ = 0.17, indicating that Han students evaluated the words with moral meaning faster than the words with immoral meaning. The main effect of the red circle position was not significant, *F*(1,29) = 2.70, *p* = 0.11, $\eta_{p}^{2}$ = 0.09. The valence × position interaction was not significant, *F*(1,29) = 0.14, *p* = 0.71, $\eta_{p}^{2}$ = 0.00.

**Table 5:** The response time of the adjusted IAT task in Han students (ms) (M ± SD).

Prime positions	Words’ moral valence	
	Moral	Immoral
Left	800 ± 100	818 ± 112
Right	793 ± 109	814 ± 112

As we mentioned previously, the association between morality and spatial metaphor, to a certain extent, is due to age. In this case, an independent-samples *t*-test of age between Hui and Han was carried out and a significant difference was found, *t*(58) = 6.78, *p* < 0.001. In order to exclude the influence of age, covariant ANOVA was adopted, while age was set as covariate, ethnic was the between-subjects variable and RTs in various conditions were independent variables. The result showed that the main effect of age was not remarkable, *F*(1,54) = 2.64, *p* = 0.08; and the interaction between age and other variables were also not significant, *F*(1,54) < 1.43, *p* > 0.25. It suggested that age could be excluded as factor influencing the result.

### Discussion

In Experiment 2, Han participants did not show a horizontal spatial bias toward moral or immoral words, equivalent to Experiment 1. This tallied with our second hypothesis. However, unlike Experiment 1, when Experiment 2 adopted the modified IAT paradigm, Hui students evaluated words with moral meaning faster when the red circle prime appeared on the right, whereas evaluated words with immoral meaning faster when the red circle prime appeared on the left, which matched our first hypothesis. It showed that Hui students were influenced by their ethnic culture, and have established horizontal spatial metaphors for morality.

## General Discussion

With two experiments, this research verified the influence of culture on horizontal spatial metaphors for morality. Both experiments selected participants from middle school students at Qutong Middle School in Qutong Village, Yongping County, Dali Bai Autonomous prefecture, Yunnan Province. Records show that the Qutong Village is a typical Islamic Hui village. It has 7,221 populations, 90% of which are of Hui ethnicity (Editorial Committee of local chronicles of Bai Autonomous Prefecture of Dali, 2014). The traditional Hui culture remains intact in this area. Therefore, following their ethnic traditions, Hui students have the idea of “right-superior and left-inferior.”

In Experiment 1, we adopted the spatial Stroop task. In this experiment, neither Han nor Hui students showed horizontal spatial bias for morality. This result partially supported our hypothesis that Han students did not establish horizontal spatial metaphors for morality, because their “left-superior and right-inferior” culture changed several times with the replacement of dynasties throughout history. However, contrary to our hypothesis, Hui students did not present horizontal spatial metaphors for morality, either. We reasoned that because the young students had weak associations between moral concepts and horizontal positions, the spatial Stroop task did not have enough strength to activate the awareness of the spatial metaphors for morality. The result further proved that the awareness of spatial position was important to determine whether Hui middle school students could develop the horizontal spatial metaphors for morality.

In Experiment 2, we adopted a modified IAT task and enhanced the spatial awareness in participants. The results of Experiment 2 showed that Hui students categorized words with moral meaning faster when the spatial prime were on the right, compared to if the prime was on the left. In contrast, they evaluated words with immoral meaning faster when the spatial primes were on the left, compare to if the prime was on the right. However, Han students did not have any bias regardless of which side the spatial primes appeared on. The results fully supported our hypothesis. That is, Hui middle school students were influenced by the “right-superior and left-inferior” culture, and have established the horizontal spatial metaphors for morality. However, since there is no stable horizontal positions for superiority and inferiority in Han culture, Han students do not form horizontal spatial metaphors for morality. Lakoff and Johnson (1980) indicated that the spatial metaphors were not formed by chance; instead, they were influenced by physical and cultural factors. The understanding toward a metaphor was also built on the culture. In our study, even though the Hui and Han participants live in the same area, their ethnic cultures are different. Therefore, the influences of culture toward their horizontal spatial metaphors for morality are divergent.

In fact, the influences of culture toward the metaphors are not limited to the horizontal spatial metaphors for morality. Tversky et al. (1991) studied the spatial metaphors for time in Jewish and Arabic children. They asked the participants to read a sequence of events in random order and link them with left-to-right arrows (e.g., power on - > input the password - > open the file). They found that Jewish children filled in events from left to right, whereas Arabic children filled in events from right to left. This disparity stems from their different writing habits. Although both Hebrew and Arabic sentences begin on the right side of the page, Hebrew words are written from left to right, whereas Arabic words are written from right to left. Therefore, the directions they write leads to different linguistic cultures for Jewish and Arabic people (Goodnow et al., 1973; Tversky et al., 1991).

In addition, both Experiments demonstrated a common phenomenon: participants responded to the words with moral meanings faster than the words with immoral meanings. We reasoned that this is due to the influence of traditional Confucian culture in China. Confucian Culture declares that “Men at their birth are naturally good.” Mencius, the most famous Confucian sage after Confucius himself, said that “The good disposition of human nature is like water’s tendency to flow down. There are no men innately bad, just as there is no water that does not flow down.” The “theory of the original goodness of human nature,” as a part of moral education in modern China (Guan, 2013), is widespread among primary and middle school students throughout the country. Therefore, in our study, both Hui and Han students evaluated words with moral meanings faster than words with immoral meanings. Wang and Lu (2013) also supported this idea by adopting a spatial Stroop task to study the vertical spatial metaphors for morality in Chinese college students. The participants evaluated the words with moral meaning significantly faster than words with immoral meaning. However, United States college students, who also evaluated words with moral meaning faster than words with immoral meaning, had no influence from Chinese Confucian culture (Meier and Robinson, 2004; Meier et al., 2007; Hill and Lapsley, 2009). Therefore, the “theory of the original goodness of human nature” can only explain the results in Chinese participants, and there are other explanations for United States college students.

Research reported “ingroup-outgroup bias” when people make moral evaluations. That is, compared to outgroups, people tended to be more tolerant toward themselves and their in-group members (Valdesolo and Desteno, 2007, 2008; Quan, 2011, unpublished). We postulated that both Chinese and United States students tended to associate the words with moral meanings with themselves when evaluating the moral valence, thus they evaluated faster for words with moral meanings.

## Author Contributions

RC design of the work; drafting the work; final approval of the version; agreement to be accountable for all aspects of the work. JS the acquisition of data and the analysis and interpretation of data for the work. QZ polish the manuscript and revising it critically for important intellectual content. RZ revising it critically for important intellectual content. PL revising it critically for important intellectual content. SH revising it critically for important intellectual content; final approval of the version to be published; agreement to be accountable for all aspects of the work.

## Conflict of Interest Statement

The authors declare that the research was conducted in the absence of any commercial or financial relationships that could be construed as a potential conflict of interest.

## Supplementary Material

DATA SHEET S1 Raw data of this research.
